# Experimental and Machine Learning Study of a Modified Cymbal Piezoelectric Energy Harvester

**DOI:** 10.3390/mi16121342

**Published:** 2025-11-27

**Authors:** Turuna Seecharan, Cobi Kiffmeyer, Nolan Voiles, Kyle Enrlichman, Alex Hankins, Ping Zhao

**Affiliations:** 1 Department of Mechanical and Industrial Engineering, University of Minnesota Duluth, Duluth, MN 55812, USA; tseechar@umn.edu (T.S.);; 2 Department of Electrical and Computer Engineering, University of Minnesota Duluth, Duluth, MN 55812, USA

**Keywords:** cymbal piezoelectric harvester, PVDF, singular value decomposition, machine learning prediction

## Abstract

Cymbal piezoelectric energy harvesters offer an effective platform for converting mechanical vibrations into electrical energy due to their ability to exploit both longitudinal (d_33_) and transverse (d_31_) piezoelectric coefficients. However, the design of flexible cymbal structures that ensure efficient stress transfer to polymer-based piezoelectric materials remains insufficiently explored. In this study, a bridge-like cymbal harvester incorporating polyvinylidene fluoride (PVDF) films as the active layer was designed, fabricated, and experimentally investigated. To support the design process and reduce the computational burden associated with evaluating multiple geometric configurations, we developed a novel machine learning methodology that integrates singular value decomposition (SVD) with metamodeling. This framework provides rapid predictions of resonance behavior and electrical response from key design parameters. The findings demonstrate the feasibility of PVDF-based cymbal harvesters for flexible energy harvesting applications and establish an efficient data-driven approach for guiding future design optimization.

## 1. Introduction

### 1.1. Piezoelectric Energy Harvesters

With the depletion of fossil fuels and the associated environmental concerns, energy harvesting devices that utilize renewable energy sources have emerged as an effective approach to mitigating global energy challenges. These harvesters can convert various forms of waste energy, such as solar, thermal, vibration, potential, and kinetic energy, into electrical energy. Among these, mechanical vibrations are particularly attractive because they are less affected by environmental conditions and can be harvested from a wide range of ambient sources, including raindrops, wind, ocean waves, human motion, machinery, and vehicles. Piezoelectric materials, which can directly convert mechanical vibrations into electrical energy, play a key role in the development of efficient energy harvesters due to their high energy conversion efficiency and suitability for integration into structural components [1,2,3,4,5,6,7].

Among various piezoelectric energy harvester configurations, cymbal-type devices have attracted considerable attention for their efficient electromechanical coupling, structural simplicity, and compact design. A typical cymbal harvester consists of a piezoelectric material sandwiched between two cymbal-shaped end caps, forming a mechanically robust and easily manufacturable structure [8,9,10,11]. The unique geometry of the cymbal structure plays a crucial role in its electromechanical performance. The cavities in the end caps function as mechanical transformers, converting and amplifying a portion of the applied axial stress into radial stress within the piezoelectric element. This stress transformation enables simultaneous utilization of both the longitudinal (d_33_) and transverse (d_31_) coefficients, effectively enhancing the overall piezoelectric response and resulting in improved power output. The performance of cymbal harvesters is strongly influenced by geometric design parameters such as cavity depth, cavity radius, and end-cap thickness [12,13,14,15,16]. Lead zirconate titanate (PZT) ceramics have been widely used as the active material in cymbal harvesters due to their relatively high piezoelectric coefficients (d_33_ = ~150 to 600 pC/N and d_31_ = ~−60 to −250 pC/N) values. Relaxor piezoelectric single crystals such as PMN-PT and PNN-PZT have also been applied, achieving even greater power output because their piezoelectric coefficients are roughly three to five times higher than those of conventional PZT ceramics [17,18]. However, the performance of single crystal cymbal harvesters is highly dependent on crystal orientation, reflecting the intrinsic anisotropy of these materials. More recently, bio-piezoelectric materials including amino-acid crystals have emerged as promising candidates for flexible and biocompatible energy harvesting due to their intrinsic sustainability, mechanical softness, and compatibility with wearable or implantable systems [19,20,21,22].

Polyvinylidene fluoride (PVDF), a piezoelectric polymer, has also been widely used in energy harvesting applications as an alternative to brittle ceramics and single crystals. Although its piezoelectric coefficients, d_33_ (~−20 to −40 pC/N) and d_31_ (~5 to 30 pC/N), are smaller than those of PZT ceramics or relaxor single crystals, PVDF offers advantages including low stiffness, high ductility, and excellent fatigue resistance. These properties make it particularly suitable for applications requiring mechanical flexibility and durability. PVDF-based energy harvesters have predominantly adopted cantilever beam configurations to capture energy from various mechanical vibrations, typically producing power outputs in the nano to microwatt range [2,23,24,25,26]. While cantilever designs remain effective, exploring alternative geometries could provide additional opportunities to leverage PVDF’s mechanical compliance and resilience. Cymbal harvesters, with their ability to sustain large deformations and cyclic loading, offer a promising but underexplored platform for PVDF integration. Therefore, in this work, we investigate the feasibility of incorporating PVDF films into a cymbal type harvester and evaluate their energy harvesting performance under mechanical excitation. Because the natural frequency of a piezoelectric energy harvester dictates the condition for maximum power generation, this study specifically focuses on the frequency-dependent voltage response to identify the resonance frequency corresponding to the highest energy output.

### 1.2. Machine Learning Approaches in Energy Harvesting

The design and optimization of vibration-based piezoelectric energy harvesters often involve complex, iterative processes, necessitating precise modeling and extensive experimental validation. Rapidly matching a harvester’s resonance frequency to ambient vibrations while maximizing power output presents a significant challenge. Traditional methods rely on analytical electro-mechanical models (e.g., coupled distributed parameter models) or finite element analysis (FEA), which can be computationally intensive and may struggle to accurately account for real-world manufacturing tolerances, material nonlinearities (especially in polymers like PVDF), and coupling effects inherent in complex geometries like the cymbal structure.

To address these challenges, machine learning (ML) approaches have been increasingly adopted and demonstrated effectiveness in optimizing energy harvesting devices [27,28,29,30]. ML algorithms provide a powerful means to model nonlinear relationships between a device’s geometric and material design parameters (e.g., thickness, length, material stiffness) and its performance metrics (e.g., resonance frequency, voltage, power). Prior studies have applied various ML techniques for this purpose, including random forest (RF) [27], artificial neural networks (ANNs) with genetic algorithms [28], layered recurrent neural networks (LRNNs) [29], and XGBoost combined with other regressors [30]. These approaches have proven capable of handling system nonlinearities, improving design efficiency, and predicting device performance more accurately than traditional methods. The study aims to integrate ML with data reduction and surrogate modeling to enhance predictive efficiency and support the systematic design of piezoelectric energy harvesters.

### 1.3. Advanced Data Reduction and Metamodeling

For systems involving high-dimensional datasets, such as those generated from frequency sweeps or numerous FEA simulations, directly training ML models can be computationally inefficient. Combining dimensionality reduction with metamodeling offers an efficient approach to capture system behavior. A metamodel is a simplified, low-cost predictive model that approximates a complex physical or analytical model. While ML broadly encompasses algorithms for learning from data, metamodeling focuses on rapid prediction and optimization, making it valuable for design space exploration and sensitivity or uncertainty analysis. Studies have applied metamodels to the design of piezoelectric energy harvesters [31,32,33]. For example, one study combined Kriging metamodels coupled with Monte Carlo simulations to design robust cantilever harvesters, revealing trade-offs between beam thickness, power output, and reliability [31]. Another used Polynomial Chaos Expansion (PCE) methods to predict electrical output efficiently, identifying piezoelectric layer length and thickness as dominant variables [32]. Moreover, Kriging models have shown strong agreement with FEA simulations, confirming their suitability for sensitivity and optimization studies [33].

Singular value decomposition (SVD) is a powerful matrix factorization technique used for model order reduction. In energy harvesting, SVD when applied to a matrix of frequency-response data (e.g., voltage output curves across a range of input frequencies and design parameters), decomposes the data matrix into orthogonal basis vectors (or modes) and corresponding singular values. The basis vectors capture dominant patterns in the frequency responses (e.g., resonance curve shapes), while the singular values indicate the importance of each mode. By retaining the modes associated with the largest singular values, the full response can be accurately represented using a significantly smaller set of modal coefficients, effectively reducing the dimensionality of the output space.

After reducing the dimensionality of the data using SVD, a ML (metamodel) algorithm approximates the relationship between design parameters (inputs) and the SVD modal coefficients (reduced outputs) rather than the full high-dimensional response curve [34]. Common metamodels include regression models, Kriging models, radial basis functions, and ANNs. This two-step process with SVD for efficient representation and ML for mapping enables rapid prediction of the entire power output spectrum for any new set of design parameters. Building on this concept, the present work introduces a novel approach that integrates SVD with metamodeling specifically for piezoelectric cymbal harvesters. By leveraging the dimensionality reduction in SVD and the predictive power of ML, the approach can directly predict key performance metrics, such as resonance frequency and voltage output, from design variables. This integration not only accelerates the design process but also enables systematic, automated optimization of the devices.

### 1.4. Paper Organization

The remainder of this paper is organized as follows. Section 2 presents the experimental design and fabrication of the modified PVDF cymbal harvester, including material selection, prototype fabrication, and voltage response measurements under controlled excitation. Section 3 outlines the modeling methodology, combining experimental data, SVD, and metamodeling to predict frequency-dependent voltage responses and resonance behavior. Finally, Section 4 concludes the study with key findings and highlights the potential of the SVD and metamodel framework for efficient design and optimization of flexible piezoelectric energy harvesters.

## 2. Experiments

To validate the proposed concept and provide data for model training, modified cymbal piezoelectric harvesters with integrated PVDF films were designed, 3D printed and tested under controlled vibration. Geometric and material parameters were varied to measure frequency-dependent voltage responses and identify the resonance characteristics of each configuration. The resulting measurements form the experimental basis for the predictive modeling described in Section 3.

### 2.1. Prototype Fabrication and Materials

**Figure 1:**
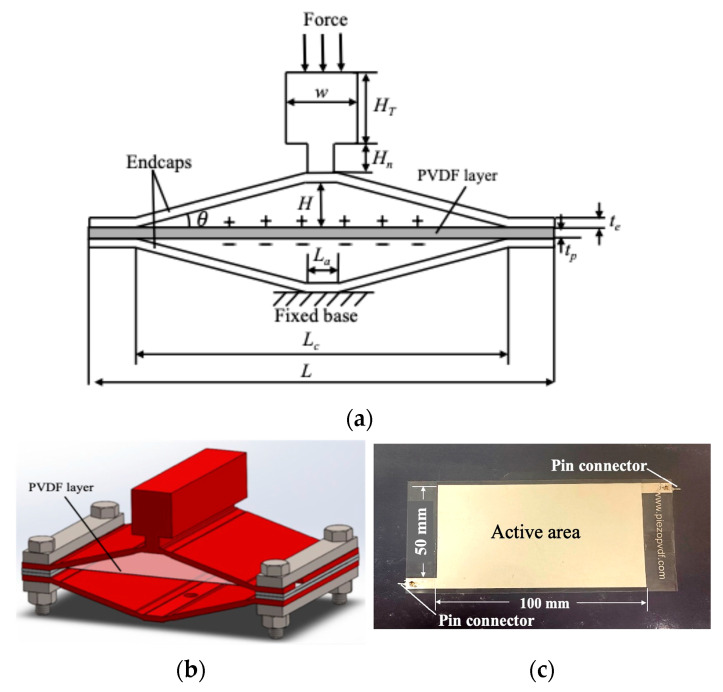
(**a**) Schematic diagram of the cross section and (**b**) SolidWorks model of the cymbal energy harvester. (**c**) Photograph of the top view of the PVDF transducer. Both sides of the PVDF active area are coated with silver electrodes, which are connected to the pin connectors.

**Figure 2:**
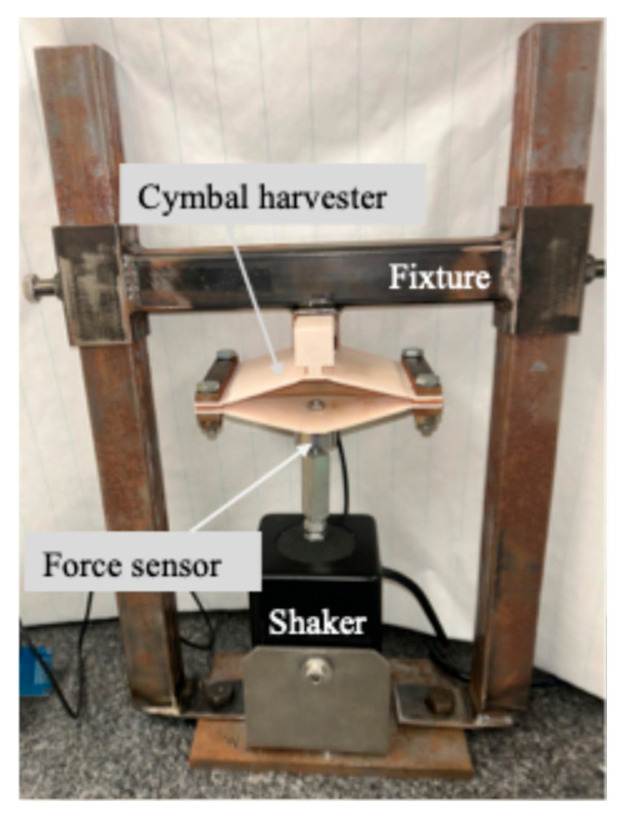
Photograph of the testing fixture mounted on a granite table. The top of the cymbal energy harvester is secured to the fixture through the T-shaped component, while the bottom of the harvester is attached to a force sensor connected to the shaker system. During testing, the top of the cymbal harvester remains fixed, and the bottom is driven by the shaker at various excitation frequencies.

While most cymbal energy harvesters use a closed circular design, rectangular configurations have also been reported for different applications [14,35,36]. In this work, a custom cymbal harvester with a modified rectangular, bridge-like geometry was designed and fabricated. Figure 1 presents (a) the schematic cross-sectional diagram and (b) the SolidWorks 2025 model of the cymbal assembly. This open structure provides direct visual and experimental access to the embedded piezoelectric layer during testing, enabling real-time observation of its deformation and dynamic response. To ensure proper clamping of the piezoelectric element, two rubber strips (1 mm thick) were positioned between the end caps and the piezoelectric layer. Holes were punched through the rubber strips to accommodate the fasteners. Steel bars (79.5 mm × 12.7 mm × 4.8 mm) were placed on the outer surfaces of the end caps to enhance clamping force while minimizing interfacial friction. This increased clamping strength allowed the prototype to sustain prolonged vibration without component slippage. A T-shaped component (width *w* = 20.1 mm, height *H_T_* = 20.1 mm, neck height *H_n_* = 5.1 mm, length *L_T_* = 79.5 mm) was incorporated at the top of the cymbal to enable insertion into the testing fixture shown in Figure 2. This design facilitates convenient assembly and disassembly of the device during experimental study.

A PVDF transducer with a thickness of 110 μm and a capacitance of ~5 nF (PolyK Technologies, State College, PA, USA) was integrated as the active piezoelectric element as shown in Figure 1c. Silver electrodes (5–10 μm thick) were screen-printed on both sides of the film, forming an active area of 100 mm × 50 mm. Electrical connections were made using two pin-type connectors attached to opposite ends of the electrode region. These pin connectors were routed to a rectifier circuit described in Section 2.2 to convert the AC voltage output into DC. The assembled transducer was laminated with a thin protective tape layer to prevent mechanical damage during handling and testing. The piezoelectric coefficients of the PVDF transducer are d_33_ = ~−33 pC/N and d_31_ = ~25 pC/N and its dielectric constant is ~11.

In previous studies, metal caps were typically employed in cymbal harvesters to withstand high mechanical loading. In the present work, which represents an initial experimental investigation, the prototypes were fabricated using 3D printing with polylactic acid (PLA) and polyethylene terephthalate glycol-modified (PETG) filaments (Bambu Lab, Shenzhen, China). This approach enabled rapid and flexible prototyping; however, it is acknowledged that the mechanical strength of polymer filaments is inherently lower than that of metals. Four prototypes with two end-cap thicknesses (2 mm and 2.5 mm) and two materials (PLA and PETG) were fabricated for this study. Table 1 summarizes the dimensions and materials of the prototypes.

### 2.2. Experimental Arrangement

**Figure 3:**
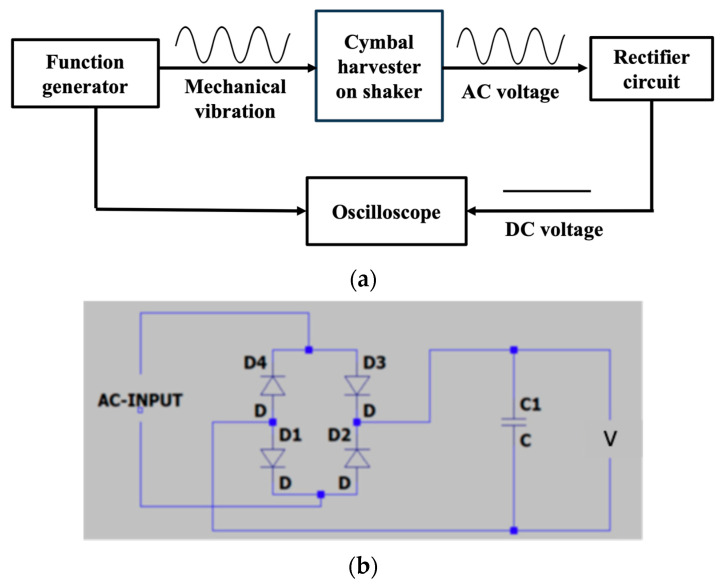
(**a**) Flow chart illustrating the testing process. (**b**) Diagram of the full bridge rectifier circuit.

For performance evaluation, the cymbal harvester was mounted on an electrodynamic shaker (Labworks LW132.151-7, Costa Mesa, CA, USA), which provided harmonic excitation at controlled frequencies and amplitudes, as shown in Figure 2. As illustrated in Figure 3a, a 200 mVp-p sinusoidal voltage was generated by a function generator (Wavetek, San Diego, CA, USA) and then sent to the shaker to simulate mechanical vibrations during testing. This input produced an RMS force of 6 N, measured by a force sensor (PCB Piezotronics, Depew, NY, USA). Under mechanical excitation, the cymbal harvester produced an AC voltage across the PVDF transducer. A full bridge rectifier circuit including four diodes (D_1_–D_4_, 1N4004, 400 V) was used to convert the AC voltage into DC, which was subsequently stored in a capacitor (C_1_, 10 μF, 450 V), as shown in Figure 3b. The rectified voltage, along with the input excitation signal, was monitored and recorded using an oscilloscope (Keysight, Santa Rosa, CA, USA). A video showing the experimental procedure has been included in the Supplementary Materials.

**Figure 4:**
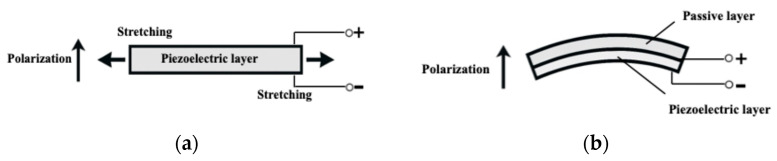
Comparison of deformation modes in piezoelectric transducers. (**a**) Extension mode: the transducer undergoes in-plane stretching, generating strain primarily along the length direction. (**b**) Bending mode: the structure deflects into a curved shape, producing compressive strain on one side and tensile strain on the other through its thickness (bending response).

PVDF transducers exhibit a much stronger response in the d_31_ (length) mode than the d_33_ (thickness) mode, meaning their signal output is significantly enhanced when stretched along the length direction (extension mode), as illustrated in Figure 4a. The PVDF film obtained from PolyK was supplied with a 125 μm-thick polyethylene terephthalate (PET) tape that was able to laminate on one surface, forming a composite structure. Since the passive PET layer was thicker than the PVDF film, the transducer primarily operated in the d_33_ (bending) mode, as shown in Figure 4b. Both extension and bending modes were experimentally investigated to compare their voltage outputs across a wide range of frequencies.

### 2.3. Experimental Results and Discussion

**Figure 5:**
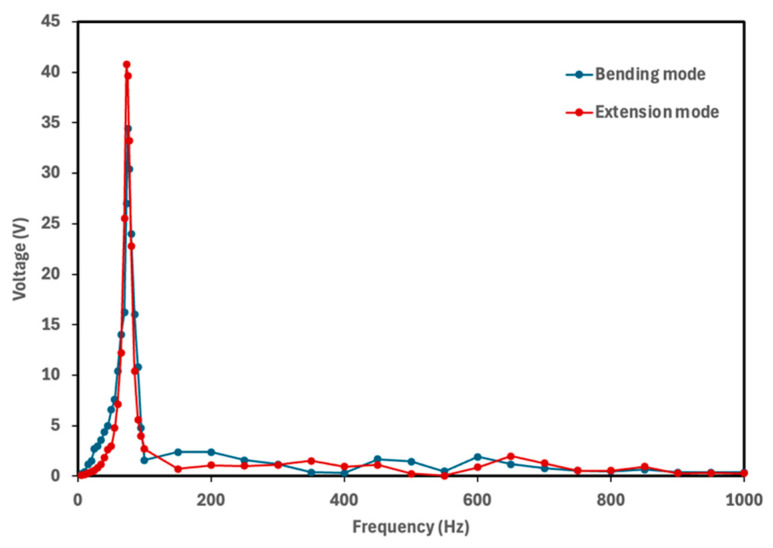
Voltage output of the 2.5 mm PLA cymbal as a function of frequency. The maximum voltage of 40 V was obtained at its resonance frequency of 73 Hz for the extension mode, while the maximum voltage of 34.4 V was obtained at its resonance frequency of 75 Hz for the bending mode.

**Figure 6:**
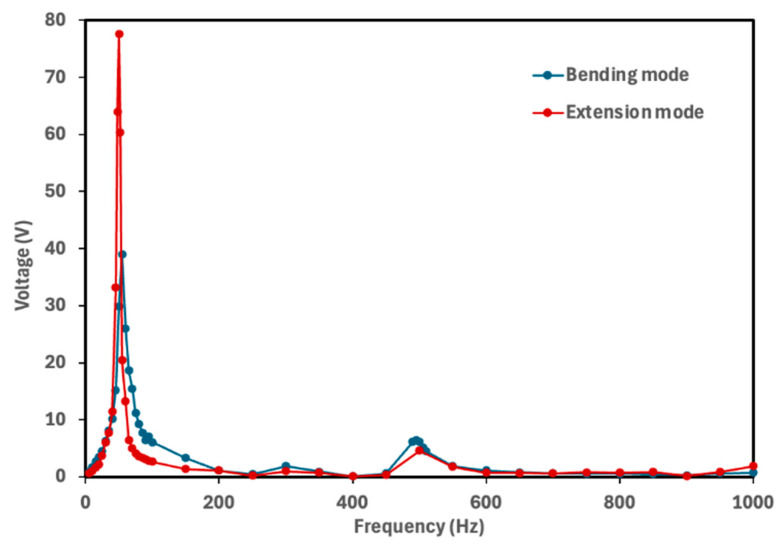
Voltage output of the 2 mm PLA cymbal as a function of frequency. The maximum voltage of 77.6 V was obtained at its resonance frequency of 50 Hz for the extension mode, while the maximum voltage of 39 V was obtained at its resonance frequency of 55 Hz for the bending mode.

**Figure 7:**
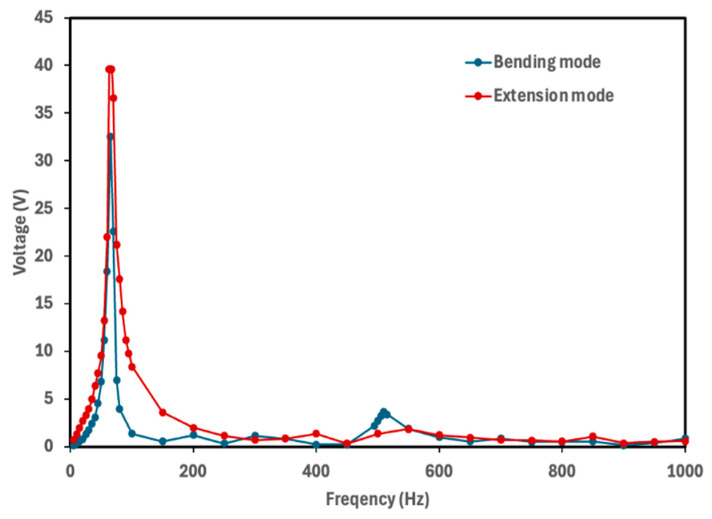
Voltage output of the 2.5 mm PETG cymbal as a function of frequency. The maximum voltage of 39.6 V was obtained at its resonance frequency of 65 Hz for the extension mode, while the maximum voltage of 32.5 V was obtained at its resonance frequency of 65 Hz for the bending mode.

**Figure 8:**
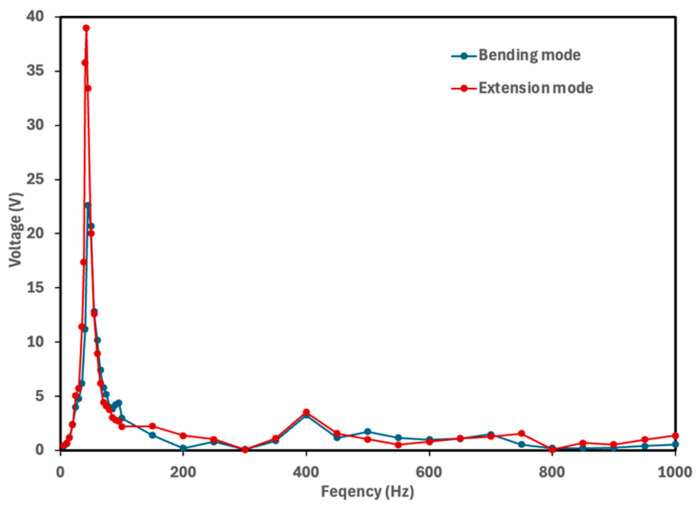
Voltage output of the 2 mm PETG cymbal as a function of frequency. The maximum voltage of 39 V was obtained at its resonance frequency of 42 Hz for the extension mode, while the maximum voltage of 22.6 V was obtained at its resonance frequency of 45 Hz for the bending mode.

The natural frequency of a piezoelectric energy harvester determines the frequency at which the device generates maximum electrical power. To identify the frequency corresponding to the maximum voltage output, each cymbal prototype was tested over a frequency range of 5–1000 Hz with finer resolution near the resonance frequencies. The rectified voltage was measured across the 10 μF capacitor. Figure 5, Figure 6, Figure 7 and Figure 8 show the voltage output of cymbal prototypes as a function of frequency. The resonant frequencies and the corresponding voltage outputs of all prototypes were successfully identified in both extension and bending modes. Devices operating in extension mode generate higher voltage outputs because the induced strain along the length direction is greater than that along the thickness. For prototypes made from the same material (PLA or PETG), the 2 mm thick cymbals produced higher voltage than the 2.5 mm ones, consistent with previous studies showing that thinner cymbals yield greater output under small loads [12,14,15,16]. This is mainly due to the higher mechanical compliance of the thinner end caps, which deform more easily and transfer strain more effectively to the piezoelectric layer, enhancing activation of the d_31_ mode. In contrast, the thicker end caps distribute stress more uniformly, resulting in lower strain in the piezoelectric film and reduced voltage output. Under large loading conditions, however, a thicker cymbal would be advantageous for its greater stiffness and load-bearing capacity, though such conditions are beyond the scope of this study. Conversely, in bending mode, the 2.5 mm prototypes produced higher voltage outputs. The thicker end cap improved load transfer to the PVDF-PET composite, inducing greater strain through the film thickness. This out-of-plane (thickness direction) strain activated the d_33_ mode, which has a higher coefficient magnitude than d_31_, leading to greater voltage generation for the 2.5 mm design.

It was also observed that the 2 mm cymbals exhibited a lower resonance frequency compared to the 2.5 mm ones, likely due to their reduced stiffness. Thinner cymbal caps are more compliant, which decreases the system’s natural frequency in accordance with classical vibration theory. Similarly to the thickness effect, the base material also influenced the voltage response of the cymbal harvesters. For prototypes with the same thickness, PLA cymbals produced higher voltage outputs than those made of PETG. This difference is mainly attributed to the higher stiffness and elastic modulus of PLA (2.75 GPa) compared to PETG (2.05 GPa). The stiffer PLA structure enables more efficient transfer of mechanical stress to the piezoelectric layer, resulting in greater induced strain and voltage generation. In contrast, PETG’s lower modulus and higher ductility lead to partial energy dissipation through viscoelastic deformation and internal damping, reducing the effective stress transmitted to the piezoelectric element and thus the voltage output.

Overall, the 2 mm thick PLA cymbal produced the highest voltage response at its resonance frequency. This can be attributed to its best combination of mechanical compliance and stress transmission efficiency to the PVDF layer. This study demonstrated the successful integration of a PVDF thin film into the modified cymbal structure. All prototypes, regardless of material or thickness, produced notable voltage outputs under low mechanical vibrations. These preliminary findings provide a solid foundation for future investigations into power performance. Because power output depends on both voltage response and load resistance, it serves as a direct measure of the device’s energy conversion capability. Future work will include systematic characterization of power behavior under various load conditions and examination of potential performance enhancement strategies, including refinement of geometric parameters, evaluation of alternative end cap materials, and assessment of improved packaging approaches to further optimize device efficiency.

## 3. Modeling

While the experimental results provided valuable insights into the performance of PVDF-based cymbal harvesters, the fabrication and testing process was both time- and resource-intensive, even with only four prototype configurations. Each specimen required individual 3D printing, assembly, and frequency-response testing, making it impractical to explore a broader design space experimentally. To overcome these limitations and accelerate future design iterations, a modeling framework was developed to efficiently predict device performance from key design variables. The experimental data obtained in Section 2 were used to train and validate this model, ensuring that the predictions are grounded in measured device behavior. The proposed approach integrates singular value decomposition (SVD) with a metamodel to capture the relationship between geometric and material parameters and the resulting voltage response. This data-driven model enables rapid estimation of resonance frequency and voltage amplitude without repeated physical testing, thereby reducing experimental workload and supporting systematic design optimization.

### 3.1. Methodology

**Figure 9:**
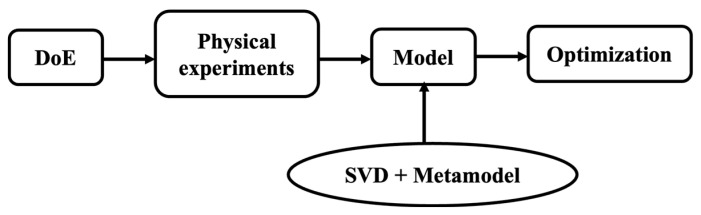
Flow chart of the proposed modeling methodology.

Building on this framework, the modeling methodology develops a simple explicit model by combining Design of Experiments (DoE) and SVD, as illustrated in Figure 9. The approach efficiently captures the relationship between design parameters (input variables such as end-cap thickness, material type and working mode) and performance metrics (output voltage responses as a function of frequency). DoE is used to select a set of experimental conditions, from which sample data are collected through physical measurements. These data form the foundation for constructing a surrogate model that integrates SVD with a metamodel, enabling accurate performance prediction and optimization with reduced computational effort. The methodology builds on previous work [34] and is implemented in the following steps.

1. A Design of Experiments (DoE) approach generates a set of *M* training datasets where each dataset corresponds to a specific combination of design parameters (*x_i_*).
2. The excitation frequency is discretized with an increment, *Δf* such that *f* = *f_1_*, *f_2_*, …, *f_N_*, where *N* represents the number of discrete frequency steps.
3. For each training case i, the voltage output, represented as *z*(*x_i_*, *f*), is recorded over the full frequency range into a data matrix, ***Z***, where each row represents one combination of design variables and each column corresponds to a discrete frequency point:

(1) $$Z=\left[\begin{array}{ccc} z(x_{1,}{\;f}_{1}) & \ldots & z(x_{1,}{\;f}_{N})\\ \vdots & \ddots & \vdots \\ z(x_{M,}\;f_{1}) & \ldots & z(x_{M,\;\;}{\;f}_{N}) \end{array}\right]$$

4. SVD then decomposes *Z* into orthogonal spaces representing the “design-variable space” *P_l_* and the “frequency space” *Q_l_*

(2) $$Z={\left[P_{l}\right]}_{M\times M}{\left[S_{l}\right]}_{M\times N}{\left[Q_{l}\right]}_{N\times N}$$

Here, *P_l_* contains the left-hand eigenvectors, *Q_l_* the right-hand eigenvectors, and *S_l_* is a diagonal matrix containing the singular values of Z in descending order.

5. The singular values in *S_l_* are partitioned into dominant and residual groups according to their magnitudes. The dominant group, denoted *S*, contains “*s*” large singular values and is retained, while the smaller values are set to zero. The value, *s*, determines how many columns of *P_l_* and *Q_l_* are required to approximate *Z* accurately. The original matrix ***Z*** can be approximated as

(3) $$Z=\left[\begin{array}{cc} P & P_{0} \end{array}\right]\left[\begin{array}{cc} S & 0\\ 0 & 0 \end{array}\right]\left[\begin{array}{c} Q^{T}\\ Q_{0}^{T} \end{array}\right]=PSQ^{T}$$

As shown in Equation (4), a new matrix, ***D***, represents the product of matrices ***P*** and ***S***

(4) D=PS

Equation (5) illustrates how to approximate the original data matrix ***Z***.

(5) $$\hat{Z}={\left[D\right]}_{M\times S\;}{\left[Q_{l}^{T}\right]}_{s\times N}$$

6. An ML model is developed for each column of ***D***. In this study, we evaluate the performance of two simple models, including linear regression and nonlinear regression (second-order polynomial) using the mean squared error (*MSE*), Equation (6), between the predicted and actual (experimental) outputs.
  (6) $$MSE=\sum_{i=1}^{N}\frac{{\left(\hat{z}\left(x_{1},f_{i}\right)-z\left(x_{1},f_{i}\right)\right)}^{2}}{N}$$
  where $\hat{z}\left(x_{1},f_{i}\right)$ represents the prediction of the voltage at a discrete frequency, $f_{i}.$

### 3.2. Results and Discussion

Following the methodology outlined in Section 3.1, we evaluated the predictive performance of the two models. Equation (7) defines the linear regression model, whereas Equation (8) represents the second-order nonlinear regression model with interaction coefficients. Using these models, the subsequent analysis compares their ability to accurately capture the relationship between design parameters and frequency-dependent voltage responses of the PVDF cymbal harvesters, providing insight into the effectiveness of each modeling approach.

(7) $$y=\beta_{0}+\beta_{1}x_{1}+\beta_{2}x_{2}+\beta_{3}x_{3}+\varepsilon$$

(8) $$y=\beta_{0}+\beta_{1}x_{1}^{2}+\beta_{2}x_{2}^{2}+\beta_{3}x_{3}^{2}+\beta_{4}x_{1}x_{2}+\beta_{5}x_{1}x_{3}+\beta_{6}x_{2}x_{3}+\varepsilon$$

In Equation (7), y is the predicted voltage, $x_{1},\;x_{2},$ and $x_{3}$ denote the design parameters, $\beta_{i}$ are the regression coefficients describing their linear effects, and ε is the model error. Equation (8) extends the model to include quadratic and interaction terms, capturing nonlinear and coupled effects among design variables. The coefficients $\beta_{i}$ quantify the strength and direction of these effects, while ε represents the residuals. The linear model provides a baseline for identifying dominant trends, whereas the second-order model accounts for complex electromechanical interactions within the cymbal harvester.

The dataset comprises the voltage outputs for eight combinations of PET-Tape, PLA, and thickness across a frequency range of 5–1000 Hz, consistent with the experimental studies. Due to the small sample size (*M* = 8), all eight training sets (Table 2) were used to train the linear and nonlinear regression models (Equations (7) and (8), respectively). The three design variables (independent variables) are Tape, PLA, and Thickness. Tape and PLA are binary indicators, where Tape = 1 corresponds to the bending mode (prototype with tape) and Tape = 0 represents the extension mode, while PLA = 1 indicates the harvester made of PLA and PLA = 0 corresponds to PETG. Thickness is a categorical variable with two levels: 2 mm and 2.5 mm. Table 2 shows the training sets, including the three design variables. As noted in the experimental section, this preliminary study aimed to assess the feasibility of integrating PVDF films into cymbal-type harvesters, resulting in a small sample size. Nevertheless, the primary objective was to evaluate the accuracy and effectiveness of the SVD with metamodel prediction framework.

The dataset matrix, ***Z***, contains the voltage recorded at 38 distinct frequencies. After SVD, three matrices as shown in Equation (9) we obtained.

(9) $$Z={\left[P_{l}\right]}_{8\times 8}{\left[S_{l}\right]}_{8\times 38}{\left[Q_{l}\right]}_{38\times 38}$$

The matrix ***Z*** can be approximated by $\hat{Z}$ as expressed in Equation (10).

(10) $$\hat{Z}={\left[D\right]}_{8\times s}{\left[Q_{l}^{T}\right]}_{s\times 38}$$

Our goal was to investigate the accuracy of fitting linear and nonlinear regression models by varying the *s* columns of matrix ***D***. Table 3 shows the performance of different *s* values when predicting the natural frequency for Tape = 1, PLA = 0, and Thickness = 2 mm (2 mm PETG cymbal prototype in bending mode) using both regression models. The nonlinear regression model yielded a lower MSE value for different *s* values, indicating a better prediction accuracy than the linear regression model. The linear regression model was only able to accurately predict the natural frequency when *s* = 8, whereas the nonlinear regression model achieved correct prediction when *s* = 4. As the number of training sets increases, the value of *s* will also increase. When comparing the estimated voltage at the natural frequency with the actual experimental measurement, the nonlinear regression had higher absolute error percentage values. Eventually, the goal is to find a model that gives good accuracy (low MSE) using minimal *s* to reduce computational time.

A similar comparison was conducted for Tape = 1, PLA = 0, and Thickness = 2.5 mm (2.5 mm PETG cymbal prototype in bending mode). Table 4 presents these results. Consistent with the previous case (Table 3), the nonlinear regression model exhibited a lower MSE value, indicating better prediction accuracy, and both models correctly predicted the natural frequency. For higher *s* values (*s* = 6 and *s* = 8), the non-linear regression model better predicted the voltage at the natural frequency. Note that the largest value of *s* is equal to the number of training sets, which in this dataset is *s* = 8.

Finally, Table 5 compares the models’ performance in predicting the case where Tape = 0, PLA = 0, and Thickness = 2.5 mm (2.5 mm PETG cymbal prototype in extension mode). Both models accurately predicted the natural frequency, while the nonlinear regression consistently exhibited a lower MSE value for all cases of *s*. Like the case presented in Table 4, the nonlinear regression model also has low error % values.

**Figure 10:**
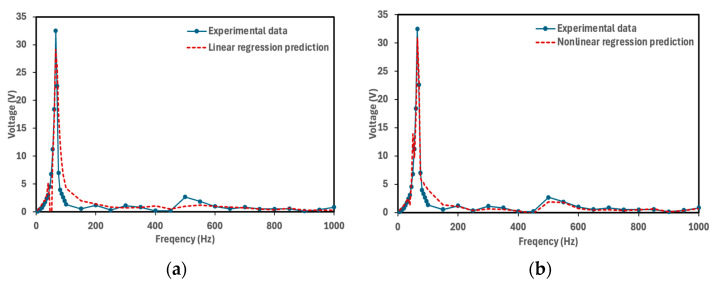
Comparison of model predictions with the experimental results for Tape = 1, PLA = 0, and Thickness = 2.5 mm (2.5 mm PETG cymbal prototype in bending mode). (**a**) Linear regression and (**b**) second-order polynomial with five singular values (*s* = 5) for predicting the voltage.

**Figure 11:**
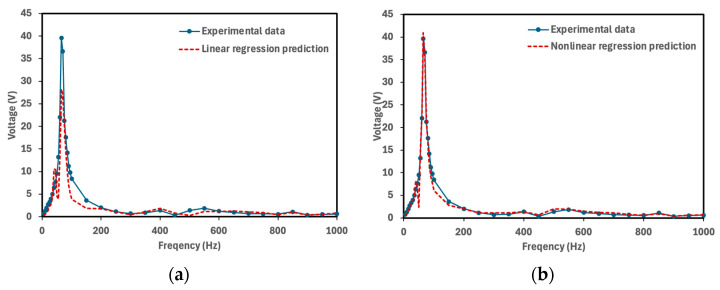
Comparison of model predictions with the experimental results for Tape = 0, PLA = 0, and Thickness = 2.5 mm (2.5 mm PETG cymbal prototype in extension mode). (**a**) Linear regression and (**b**) second-order polynomial with five singular values (*s* = 5) for predicting the voltage.

Figure 10 illustrates the predictions of the two models in comparison with the experimental results for Tape = 1, PLA = 0, and Thickness = 2.5 mm (2.5 mm PETG cymbal prototype in bending mode), while Figure 11 shows the corresponding comparison for Tape = 0, PLA = 0, and Thickness = 2.5 mm (the same prototype under extension mode). The comparison indicates that the second-order polynomial model provides better prediction accuracy than the linear model. As shown in Figure 10 and Figure 11, the nonlinear model more effectively captures the experimental trends, especially near the resonance region where the voltage response becomes strongly nonlinear.

In summary, the surrogate modeling approach that integrates SVD with a metamodel proved both efficient and accurate. The model successfully captured the voltage response and natural frequency of the cymbal PVDF harvesters under vibrational excitation even with the simplest metamodel. This hybrid approach reduced computational workload while maintaining predictive accuracy, demonstrating its practicality as a tool for rapid performance evaluation and its potential for guiding future design optimization of energy harvesting devices.

## 4. Conclusions

This study successfully demonstrated the synergistic potential of novel geometric design and machine learning in advancing piezoelectric energy harvesters. The SVD with metamodel approach effectively predicted both voltage response and natural frequency even with a simple metamodel, while significantly reducing computational workload. A 3D-printed, bridge-like cymbal harvester with PVDF transducers was introduced, effectively exploiting the d_33_ and d_31_ coupling mechanisms. Frequency-dependent voltage responses were experimentally investigated under mechanical excitation from 5 to 1000 Hz. All prototypes, operating in extension and bending modes, generated notable voltage outputs at resonance, with the 2 mm PLA prototype achieving the highest peak due to its optimal combination of mechanical compliance and efficient stress transmission. These results confirm the high energy conversion efficiency inherent to the cymbal geometry, highlighting its suitability for flexible applications. Although validated using PVDF, the design principles and ML methodology can be extended to other piezoelectric polymers with similar electromechanical characteristics by updating the material parameters in the model.

The predictive capability of the SVD and metamodel framework provides a foundation for future work, including evaluating power performance under resistive loading, exploring flexible packaging solutions, and scaling through array configurations. Future modeling efforts will evaluate other metamodels, like Kriging; expand datasets to include additional design variables, including geometric design and material properties, and allow for separate training and testing datasets; and incorporate sensitivity analysis to identify the most influential parameters. Overall, integrating machine learning with experimental validation offers an efficient and practical approach for predicting device performance and guiding the design of high-performance piezoelectric energy harvesters.

## Figures and Tables

**Table 1:** Materials and dimensions of the cymbal prototypes.

Materials and Dimensions	Prototype 1	Prototype 2	Prototype 3	Prototype 4
Material	PLA	PETG	PLA	PETG
Endcap thickness (mm) *t_e_*	2.0	2.0	2.5	2.5
Endcap angle (°) *ϴ*	15			
Cymbal total length (mm) *L*	130			
Cymbal width (mm) *W*	79.5			
Cavity height (mm) *H*	12.5			
Cavity length (mm) *L_c_*	102			
Apex length (mm) *L_a_*	9.0			
PVDF thickness (μm) *t_p_*	110			

**Table 2:** Training sets including three design variables (Tape, PLA, and Thickness).

*M* (Training Sets)	Tape	PLA	Thickness (mm)
1	1	1	2.5
2	1	1	2.0
3	1	0	2.5
4	1	0	2.0
5	0	1	2.5
6	0	1	2.0
7	0	0	2.5
8	0	0	2.0

**Table 3:** Model performance comparison in estimating the natural frequency and the voltage at the natural frequency for Tape = 1, PLA = 0, and Thickness = 2 mm (2 mm PETG cymbal prototype in bending mode). Experimental natural frequency = 45 Hz and voltage at the natural frequency = 22.6 V.

Number of Singular Values *s*	Linear Regression				Nonlinear Regression			
	MSE	Natural Frequency (Hz)	Voltage at Natural Frequency (V)	Voltage Error %	MSE	Natural Frequency (Hz)	Voltage at Natural Frequency (V)	Voltage Error %
4	8.02	50	24.83	9.87	2.36	45	18.28	−19.12
5	8.14	50	24.23	7.21	2.51	45	17.740	−21.5
6	8.07	50	24.23	7.21	2.63	45	17.72	−21.59
7	8.10	50	24.25	7.30	2.41	45	18.23	−19.34
8	7.60	45	23.92	5.84	1.72	45	21.01	−7.04

**Table 4:** Model performance comparison in estimating the natural frequency and the voltage at the natural frequency for Tape = 1, PLA = 0, and Thickness = 2.5 mm (2.5 mm PETG cymbal prototype in bending mode). Experimental natural frequency = 65 Hz and voltage at the natural frequency = 32.5 V.

Number of Singular Values *s*	Linear Regression				Nonlinear Regression			
	MSE	Natural Frequency (Hz)	Voltage at Natural Frequency (V)	Voltage Error %	MSE	Natural Frequency (Hz)	Voltage at Natural Frequency (V)	Voltage Error %
4	13.40	65	28.09	−13.58	3.50	65	27.35	−15.84
5	12.61	65	29.10	−10.47	2.40	65	30.85	−5.06
6	12.78	65	29.16	−10.29	2.19	65	30.76	−5.37
7	12.56	65	29.64	−8.79	1.83	65	31.94	−1.72
8	12.45	65	29.90	−8.00	1.72	65	32.24	−0.81

**Table 5:** Model performance comparison in estimating the natural frequency and voltage at the natural frequency for Tape = 0, PLA = 0, and Thickness = 2.5 mm (2.5 mm PETG cymbal prototype in bending mode). Experimental natural frequency = 65 Hz and voltage at the natural frequency = 39.6 V.

Number of Singular Values *s*	Linear Regression				Non-Linear Regression			
	MSE	Natural Frequency (Hz)	Voltage at Natural Frequency (V)	Voltage Error %	MSE	Natural Frequency (Hz)	Voltage at Natural Frequency (V)	Voltage Error %
4	15.59	65	24.00	−39.39	3.790	65	38.840	−1.92
5	14.21	65	28.24	−28.68	2.275	65	40.932	3.36
6	14.34	65	28.20	−28.79	2.039	65	41.038	3.63
7	14.24	65	27.93	−29.46	1.826	65	40.157	1.41
8	14.18	65	27.58	−30.50	1.724	65	39.864	0.67

## Data Availability

The data presented in this study are available on request from the corresponding author. The data are not publicly available due to privacy restrictions.

## Supplementary Materials

The following supporting information can be downloaded at: https://www.mdpi.com/article/10.3390/mi16121342/s1, Video S1: Experimental procedure.

## Abbreviations

The following abbreviations are used in this manuscript:

PVDF	Polyvinylidene fluoride
SVD	Singular value decomposition
PZT	Lead zirconate titanate
ML	Machine learning
FEA	Finite element analysis
RF	Random forest
ANN	Artificial neural networks
LRNN	Recurrent neural networks
PCE	Polynomial chaos expansion
PLA	Polylactic acid
PETG	Polyethylene terephthalate glycol-modified
PET	Polyethylene terephthalate
DoE	Design of experiments
